# Supplementary material for: Natural Coumarin Shows Toxicity to Spodoptera litura by Inhibiting Detoxification Enzymes and Glycometabolism
Source: Int J Mol Sci. 2023 Aug 24;24(17):13177. doi: 10.3390/ijms241713177 (PMC10488291; doi:10.3390/ijms241713177)

Figure S1. Identification of DEGs from 0 h to 24 h and 24 h to 48 h in *S. litura* after coumarin treatment. The red and green points represent upregulated genes and downregulated genes, respectively. The blue points indicates no significant difference.

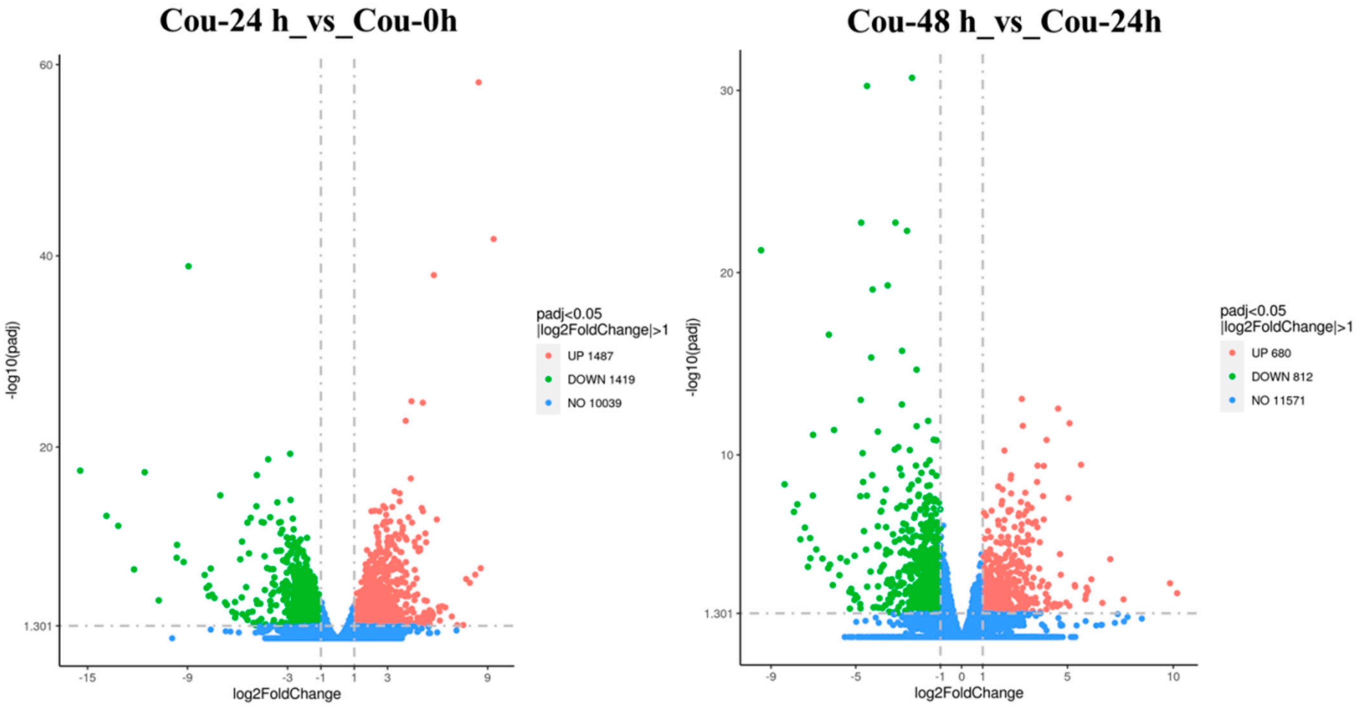

Supplement: Supplementary file 1 [file ijms-24-13177-s001.zip › Figure S1.pdf]
