# Supplementary material for: Natural Coumarin Shows Toxicity to Spodoptera litura by Inhibiting Detoxification Enzymes and Glycometabolism
Source: Int J Mol Sci. 2023 Aug 24;24(17):13177. doi: 10.3390/ijms241713177 (PMC10488291; doi:10.3390/ijms241713177)

Figure S2. Hierarchical cluster analysis of differentially expressed genes (DEGs) at 0 h, 24 h and 48 h in *S. litura* after coumarin treatment. Columns indicate different samples. Rows represent different DEGs. Green bands indicate a low expression level, and red bands indicate a high gene expression level.

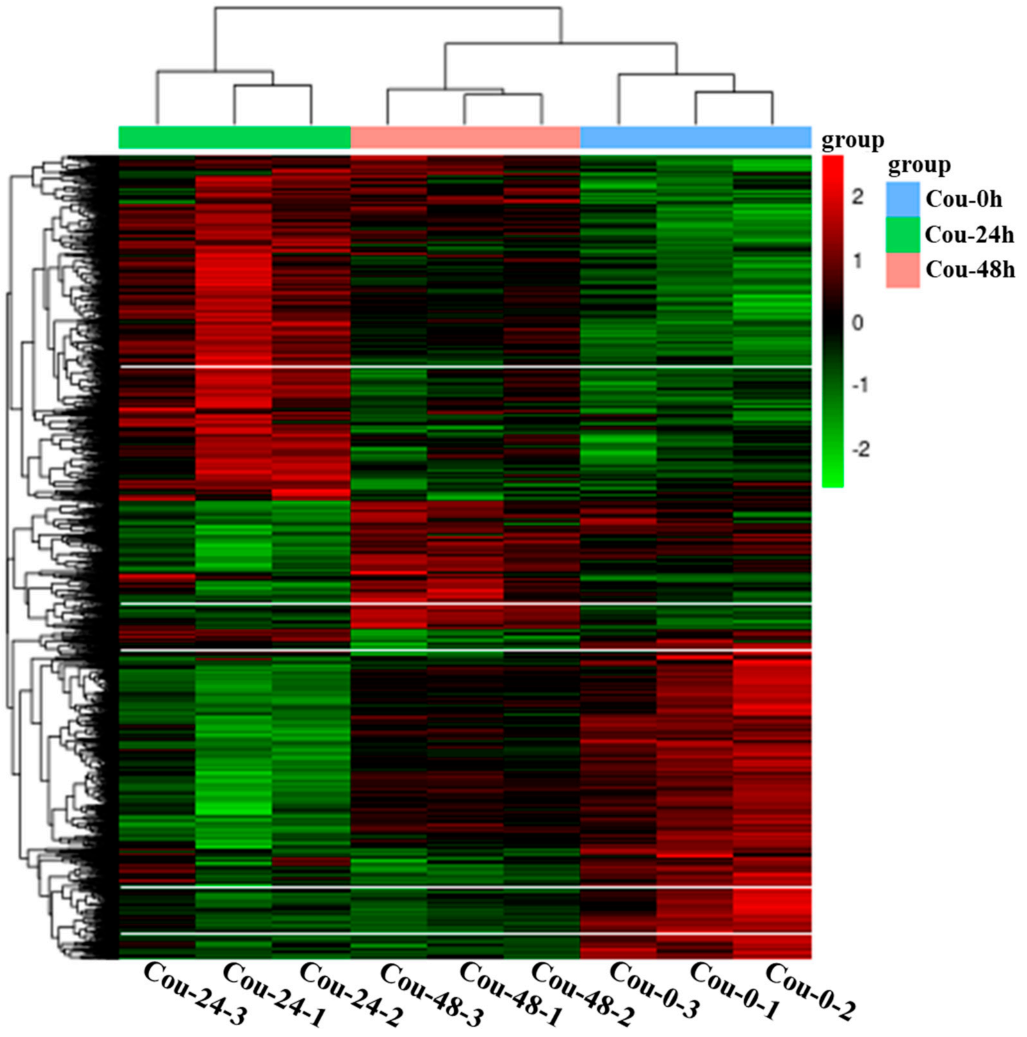

Supplement: Supplementary file 1 [file ijms-24-13177-s001.zip › Figure S2.pdf]
