# Supplementary material for: Natural Coumarin Shows Toxicity to Spodoptera litura by Inhibiting Detoxification Enzymes and Glycometabolism
Source: Int J Mol Sci. 2023 Aug 24;24(17):13177. doi: 10.3390/ijms241713177 (PMC10488291; doi:10.3390/ijms241713177)

Figure S3. GO enrichment analysis of DEGs from 0 h to 24 h and 24 h to 48 h in *S. litura* after coumarin treatment. The size of the bubble indicates the number of DEGs enriched to the corresponding term. The color of the bubble indicates the Q value.

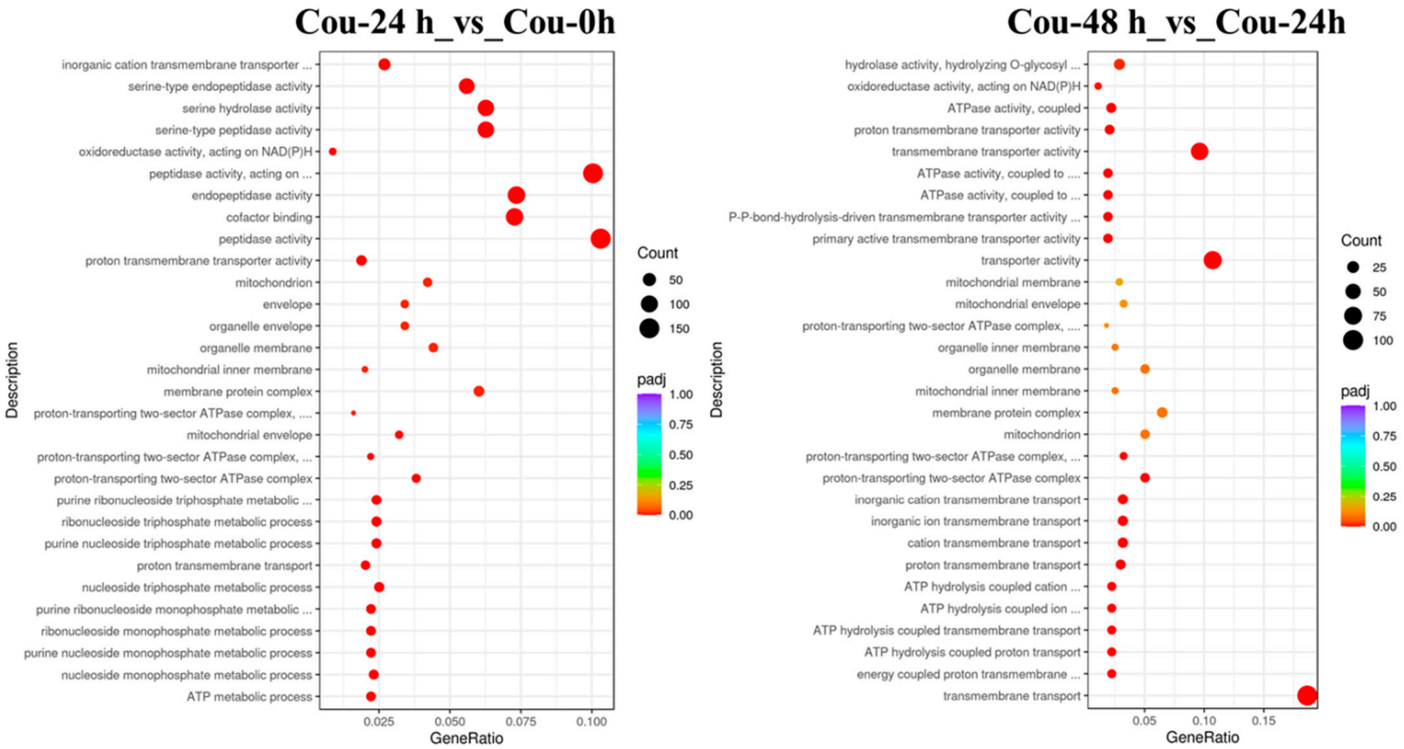

Supplement: Supplementary file 1 [file ijms-24-13177-s001.zip › Figure S3.pdf]
