# Supplementary material for: Natural Coumarin Shows Toxicity to Spodoptera litura by Inhibiting Detoxification Enzymes and Glycometabolism
Source: Int J Mol Sci. 2023 Aug 24;24(17):13177. doi: 10.3390/ijms241713177 (PMC10488291; doi:10.3390/ijms241713177)
Supplement: Supplementary file 1 [file ijms-24-13177-s001.zip › Table S1.pdf]

Table S1. Primers used in this study.

Primers	Sequences	Purpose
Venom-COE	CGTCTACACGCCGATACTGAAC<br>GGGAGCGTAAGCATCACCAG	
Cholinesterase 1	ACCTGTGCCTGATGGGTCTTC<br>ATGTCATCCCAGAAGCGTGTG	
Esterase B1	GCCCGTAGCACGAGCATTTA<br>CGTCCGTTGTCCTTGTTTGG	
AChE1	GGTGGTGCTATTGGTGTCGTT<br>ATCAAATCCTGGGTGCGGTAT	
GST-like	CTGGCTATCGCAAAGTATGTGG<br>CGCATAGGACAAACTCCACCC	
LOC111354038	CTATCAATCGCTCGCTACCTCG<br>TCCTTCACAGCCTCGTCCTTT	RT-qPCR analysis
GST-1a	AAATCCTCTCCACACAGTCCCT<br>AAAAGCCCGTTTCTTGACATCT	
GST-1b	CACAAGTCCCCTGAGTTCAAAAA<br>GTGTTGGGCTGTTGGCTTGTAT	
P4509e2	GGCGTAGCCCTTGACAGACT<br>CGGTGGATTGGGTAAACTGG	
P450304a1	CCGTCCCGAAAGATTCCTCAA<br>GGAATGGTGGTGATGATGCCT	
P4506B6	AAAAGTAAATCCCGATGGCAGT<br>TTTCGCCAACACCCTGTCTAT	
CYP324A16	CCCCAAAGACACGATGAAGC<br>AGCCACCCAGCAAGAACACT	
GAPDH	GGGTATTCTTGACTACAC<br>CTGGATGTACTTGATGAG	
dsP4506k1	TAATACGACTCACTATAGGCCTCATTATCCTCCACATCCC<br>TAATACGACTCACTATAGGAACGCTGATTCTCCAATGACA	dsRNA synthesis
dsGFP	TAATACGACTCACTATAGGCAGTGCTTCAGCCGCTACCC<br>TAATACGACTCACTATAGGACTCCAGCAGGACCATGTGAT	

The underline represents the T7 promoter sequence.
